# Empirical determination of sustainable withdrawal rates considering historical yields and inflation rates in Germany

**DOI:** 10.1007/s12297-021-00504-1

**Published:** 2021-09-24

**Authors:** Alexander Dziwisch, Philippe Krahnhof, Alexander Zureck

**Affiliations:** 1 FOM Hochschule für Oekonomie & Management gGmbH, Herkulesstraße 32, 45127 Essen, Germany; 2 Department Finance at Faculty of Economics and Administration, Masaryk University, Brno, Czechia

## Abstract

On account of the current low interest rate phase, which is most likely to continue in the coming years, the average yields to be achieved in the bond, time deposit and savings product sectors are declining, so that risk-averse investors in particular have few opportunities to generate return-oriented retirement provisions.

This scientific article analyzes the level of a possible safe withdrawal rate for diversified pension portfolios, considering historical returns and inflation rates. Consequently, this article provides immediate practical added value for a possible retirement provision.

The evaluation is based on the consideration of historical returns of the stock and bond market in Germany. To determine a safe withdrawal rate, the development of portfolios with different compositions and inflation-adjusted withdrawal rates are simulated over periods of 15 to 35 years. In this simulation, the risky part of the portfolio is represented by German equities, the low risk part by German government bonds.

To sum up, the empirical results show a maximum safe withdrawal rate of 4%. The underlying portfolio is composed of 50% equities and 50% government bonds. Particularly due to the outlined demographic change in Germany as well as the ongoing low-interest phase, the empirical study can provide significant theoretical and practical insights.

**Supplementary Information:**

The online version of this article (10.1007/s12297-021-00504-1) contains supplementary material, which is available to authorized users.

## Abstract (de)

Aufgrund niedriger Geburtenraten verbunden mit der durchschnittlich ansteigenden Lebenserwartung sank das Rentenniveau netto vor Steuern in den letzten dreißig Jahren auf rund 48 %. Durch die momentane Niedrigzinsphase, welche auch in den kommenden Jahren anhalten wird, sinken die durchschnittlich zu erzielenden Renditen im Anleihen‑, Termineinlagen- und Sparproduktbereich, sodass insbesondere risikoaverse Anleger wenige Möglichkeiten haben, renditeorientierte Altersvorsorge zu betreiben.

Der vorliegende wissenschaftliche Beitrag analysiert, in welcher Höhe eine mögliche sichere Entnahmerate für diversifizierte Rentenportfolios unter Berücksichtig historischer Renditen und Inflationsraten ausfallen kann. Folglich liefert dieser Artikel unmittelbaren praktischen Mehrwert für die Planung privater Altersvorsorge in Deutschland. Folglich liefert dieser Artikel relevante Erkenntnisse die Versicherungsbranche.

Die Auswertung basiert auf der Betrachtung historischer Renditen des Aktien- und Anleihenmarktes in Deutschland. Um eine sichere Entnahmerate zu ermitteln, wird die Entwicklung von Portfolios mit unterschiedlichen Zusammensetzungen und inflationsangepassten Entnahmeraten über Zeiträume von 15 bis 35 Jahren simuliert. Hierbei wird der risikobehaftete Teil des Portfolios durch deutsche Aktien, der risikolose Teil durch deutsche Staatsanleihen dargestellt.

Resümierend ist zu konstatieren, dass die Empirie eine maximale sichere Entnahmerate von 4 % ermittelt. Das zugrundeliegende Portfolio setzt sich aus 50 % Aktien und 50 % Staatsanleihen zusammen. Insbesondere aufgrund des skizzierten demografischen Wandels in Deutschland sowie der anhaltenden Niedrigzinsphase kann die Empirie signifikante theoretische und praktische Erkenntnisse liefern.

## Introduction

Low birth rates and increasing life expectancy have led to a demographic change taking place in Germany. Consequently, the net pre-tax pension level has fallen from 55.0% in 1990 to 48.2% in 2020 (Bundesministerium für Arbeit and Soziales 2019). Currently, no statutory lower limit is envisaged for the development of the pension level from 2030 (Deutsche Rentenversicherung Bund 2019). Even if the pension is secured by intergenerational contract, according to current forecasts, this alone will not be enough for the majority of the population to maintain their standard of living in retirement (Goebel and Grabka 2011, pp. 101–118). The risk of poverty in old age outlined above is further exacerbated by the financial situation on the money and capital markets, as the current yields on call money, fixed-term deposits or checking accounts, as well as savings accounts, are around 0% due to the loose monetary policy of the European Central Bank. As a result, German households are increasingly investing in the capital market and in some cases moving away from traditional forms of investment such as real estate, life insurance and home savings contracts. Nevertheless, the proportion of shares held by Germans in old-age provision is only a small proportion. For example, the current shareholder share of the German population is a low 15.2% (Deutsches Aktieninstitut 2019a).

Regardless of the amount of assets available at retirement and how they are distributed, a fundamental question arises for every retiree: what amount, or rather what percentage, can regularly be withdrawn from invested assets? A sustainable withdrawal strategy is necessary to avoid using up the accumulated assets within an expected lifetime.

The aim of the article is to determine an appropriate withdrawal rate at retirement age of 67 from the accumulated assets. Consequently, the paper is dedicated to the determination of a historically safe withdrawal rate for of an individually compiled portfolio consisting of shares and bonds.

## State of research

Most published studies relate to the U.S. In the U.S., the so-called 4% rule has become established as a benchmark for answering the question of the ideal withdrawal rate. This rule states that 4% of the original portfolio value can be withdrawn annually from portfolios with half the weighting of equities and half the weighting of government bonds, adjusted for inflation, without the assets being depleted within 30 years (Bengen 1994; Lucius and Lucius 2016, pp. 317–343). Historically, all portfolios in the U.S. with a 50/50 distribution of stocks and bonds have survived each 30-year period at an inflation-adjusted withdrawal rate of 4% (Bengen 1994).

The following quote from Nobel Prize winner William F. Sharpe is provided as an example of existing inefficiencies of the 4% rule:

The 4% rule and its variants finance a constant, non-volatile spending plan using a risky, volatile investment strategy. Two of the rule’s inefficiencies—the price paid for funding its unspent surpluses and the overpayments for its spending distribution—apply to all retirees, independent of their preferences. (Scott et al. 2009, p. 44)

These and other criticisms have led to many publications that do not reach a consensus in recommending sustainable withdrawal rates.

The studies can basically be distinguished according to two applied research methods. The first work is based on the observation of real, historical returns, which are tested in overlapping or rolling periods with different withdrawal rates (Bengen 1994, 1996). In order to account for historically unobserved but possible less favorable return sequences, a second approach has been the implementation of Monte Carlo simulations (Pye 2000). In a study comparing these computational methods, the authors conclude that neither method appears to be superior to the other. Depending on the parameters, the results produced can be similar or very different (Cooley et al. 2003, p. 115).

Most of the literature considers equities and bonds as asset classes of fixed income portfolios. As time goes on, research papers consider e.g. indices and investment strategies as alternative sources for both asset classes. For example, long-dated corporate bonds (Cooley et al. 1998) and inflation-linked government bonds (Pye 2000) are used instead of government bonds. For example, instead of the return of the entire U.S. stock market, small cap stocks are tested (Bengen 1997), the 4% rule is extended to include international diversification, and value and growth strategies are tested (Guyton 2004).

There is also no consensus in the literature on the minimum accepted probability of portfolio success. For example, some authors consider the recommendation of a withdrawal rate acceptable already at a historical success rate of 75% (Cooley et al. 2003, p. 127, 2011, p. 48). Although in this constellation historically every fourth portfolio has failed, the position is justified with the assumption that the withdrawal can be adjusted to the current market development. In contrast, other authors consider it a great risk to accept a withdrawal rate with lower success rates than 94% (Spitzer et al. 2007, p. 58), respectively 95% (Terry 2003, p. 65). Most statements probably lie between the positions mentioned but tend to be well above the 90%. However, there is a broad consensus that the definition of an “acceptable” success rate is a characteristic to be determined individually.

By strictly following the original, static 4% rule, investors have no flexibility with respect to changing market and portfolio developments or changing life circumstances. In academic discourse, dynamic approaches have emerged that address this circumstance. A large portion of these studies examine withdrawal strategies that take portfolio performance into account in the withdrawal rate (Mitchell 2011). For example, the static withdrawal rate is made more flexible by market-dependent upper and lower limits (Bengen 2001; Guyton and Klinger 2006). Or the adjustment of withdrawal rates structured by defined decision rules (Guyton 2004; Guyton and Klinger 2006). Other works model dynamic withdrawal rates considering remaining life expectancy (Dus et al. 2005; Milevsky and Huang 2011) or consider tax aspects (Dammon et al. 2004). High importance is also given to studies that examine the implication of longevity and mortality risks on withdrawal rates (Bodie 2004; Lachance 2012).

Typically, the recommendation for retirees has been to retire with bonds because of the low risk, or to gradually increase the proportion of bonds as they age. However, several studies conclude that this arrangement is suboptimal and in some cases the opposite is true (Basu and Drew 2009). After a conservative start with a high bond share, an increasing share of stocks towards the end lowers the risk of the portfolio going bankrupt (Shiller 2005). Other work confirms this and empirically shows that a 60/40 equity/bond allocation, is close to optimal (Kitces and Pfau 2014, p. 19; Estrada 2016). The results of the bulk of the work suggest that the equity portion of a bond portfolio should be at least 50%.

In small steps, a study visualizes the relationship between increasing withdrawal rate and increasing default risk. Here, with a 50/50 split of stocks and bonds and a withdrawal rate of 4.4%, the probability of default is 10% (Spitzer et al. 2007). Another Monte Carlo simulation arrives at a safe withdrawal rate of only 2.52% and shows that the probability of failure of the 4% rule has so far been significantly underestimated at 18% (Athavale and Goebel 2011). However, the calculated withdrawal rates in research papers are always a result of the model parameters applied. Most results on withdrawal rates are in the range of 3.5 to 4.5% for risk averse investors. With existing risk tolerance, which is measured by the success rate, this value increases to approx. 5–7% (Finke et al. 2012, p. 44). The weighting of the asset classes is crucial and cannot be applied to every portfolio constellation. The examples of recommended withdrawal rates listed apply to portfolios with an equity weighting of 50 to 75%. In very few studies can unrestrictedly comprehensible recommendations for higher or lower equity weightings be found.

Scholarly discussion of the 4% rule has long focused on the U.S. capital markets but has been extended internationally by a 17-country study (Estrada 2018). Here, the 4% rule remained in place for certain compositions in only four countries over the period 1900–2008. Germany was not one of them.

In summary, from today’s perspective, the 4% rule can be interpreted as clearly overoptimistic and has been overtaken by scientific progress (Pfau 2017). However, despite extensive research, 25 years of research have not succeeded in developing a generally valid and accepted withdrawal rate or strategy. The fact that individual investment, pension, estate, and tax aspects must be considered for each person illustrates the complexity of the issue (Sharpe et al. 2007, p. 1). The “4% rule” based on this research result has become established as a “rule of thumb” among financial planners in the USA. Critics emphasize that this is a gross simplification of complex interrelationships. Thus, adjustments to the 4% rule are needed.

Because public pensions in the U.S. are only one pillar for basic retirement security, the number of financial planners in the U.S., who hold the title of Certified Financial Planner (CFP), among others, is increasing. Also because of tax incentives for private and company pension plans, the topic of retirement planning has a higher priority in the U.S. than in Germany. This is one of the main reasons why research on withdrawal strategies is largely influenced by U.S. studies.

In research on withdrawal strategies and safe withdrawal rates, the German capital market has been considered in only a few studies to date. Due to decreasing pension benefits as well as an increasing withdrawal period, the social interest in pension development is growing. For this reason, this paper can provide both academic and practical added value.

## Empirical approach

Two main objectives are pursued in this paper. The first objective is to determine which maximum withdrawal rate has been safe in the context of historical returns and inflation rates or whether the 4% rule can also be applied in Germany. The second objective is to investigate which weighting of equities and bonds is optimal for retirement portfolios in terms of risk return. The central guiding questions established from the formulated objectives are as follows:

1. What inflation-adjusted rate can regularly be withdrawn from a diversified retirement portfolio consisting of German equities and government bonds without completely depleting the assets within 30 years?
2. What is the advantage of high bond weightings in a diversified retirement portfolio?
3. What is the advantage of high equity weightings in a diversified retirement portfolio?

This study is based on historical returns of the stock and bond market in Germany. To determine a safe withdrawal rate, the development of portfolios with different compositions and inflation-adjusted withdrawal rates is simulated over periods of 15 to 35 years. The risky part of the portfolio is represented by German equities, the risk-free part by German government bonds. The concept of the study is largely adopted from the study known as the “Trinity Study” (Cooley et al. 1998).

The Trinity Study goes back to three professors at Trinity University in Texas in 1998. In this study, a portfolio consisting of 60% equities and 40% bonds was modeled, which generated an annual return of around 4% and compensated for inflation. Details are given in the chapter “Methodology and Model Assumptions” (Cooley et al. 1998).

## Data basis of the risky part of the portfolios—equities

For the simulation of the annual return of the German market portfolio, the Frankfurt Top Segment Series (FTS Series) by Stehle/Hartmond and Stehle/Schmidt (Stehle and Hartmond 1991) was used. It represents a solid, historical database of the Prime Standard as the highest stock exchange segment of companies listed on the Frankfurt Stock Exchange. The series was compiled from various official sources after extensive review and verification for the years 1954 to 2013 (Stehle and Schmidt 2015). The developments of the New Market are not considered in the calculations of this series. In order to meet the requirement of determining the total return from the perspective of a German investor, this data series also takes into account the latter corporate income tax credits in the years 1977 to 2000 in the return calculation, in addition to normal and special dividends, capital increases, subscription right proceeds and par value conversions. However, as this series ended in 2013, whilst showing a (geometric) median return difference of only 0.003% in the years 2004–2013 in comparison to the CDAX performance index, the CDAX performance index is used as the proxy for the German capital market. With currently 485 stocks, the CDAX is significantly broader based than the DAX.

In contrast, the FTS series calculates the return on the market portfolio consistently by market capitalization over the entire period up to and including 2013. This leads to the assumption that the return of the FTS series is distorted compared to the CDAX from 2002 onwards due to the calculation method. However, since the aftermath of the dissolution of the “New Market” was only fully completed in 2003, the two indices should be compared in the years 2004 to 2013. As can be seen in Table 1: Deviations of geometric means of FTS and CDAX, the geometric mean of the annual returns of both data sets in the period 2004 to 2013 is almost identical at around 9.48%, with the deviation amounting to only 0.003 percentage points. This is to be seen as a clear difference to the deviation in the period 1970–2003 (Stehle and Schmidt 2015, pp. 440–441). Therefore, the data series of the official CDAX performance index from the year 2004 onwards will serve as the basis for the return calculation of the German market portfolio in this study.

**Table 1:** Deviations of geometric means of FTS and CDAX

Period	Geom. mean FTS (%)	Geom. mean CDAX (%)	Abs. difference FTS vs. CDAX (%)
1970–2003	8.491	7.060	1.431
2004–2013	9.482	9.479	0.003
1970–2013	8.715	7.605	1.110

Source: Own presentation. Results are rounded to the third decimal place

## Data basis for inflation values

To show the loss of purchasing power, inflation rates in Germany published by the Federal Statistical Office are used.

## Methodology and model assumptions

The focus of this paper is on the results on different withdrawal rates, which are simulated using historical returns. The methodology used for this purpose is based on two existing studies that have significantly shaped the research on withdrawal strategies (Bengen 1994; Cooley et al. 1998). Since publication, Monte Carlo simulations have been added to the methodology, but this paper will use the original rolling time period methodology for better comparability with results from the Trinity study (Cooley et al. 1998).

The composition of the portfolios is simulated with variable risk profiles. Portfolio composition is staggered in 25% increments, starting with 100% bonds and 0% stocks and ending with 0% bonds and 100% stocks. Withdrawal rates between 3 and 9% are tested. Tax effects are not considered in this model calculation, as these are personal and individual parameters. The obvious approach of using the TER (Total Expense Ratio) of a current, market-wide ETF on the CDAX as a cost factor for custody account fees must be rejected, as ETFs did not exist in 1955 and, moreover, management fees before the Internet era were most likely very different from today’s cost structures. Transaction costs have also changed continuously over time and are difficult to determine historically. Thus, considering a flat transaction cost rate for portfolio reallocations is difficult, in part because reallocations fluctuate due to variable weightings and returns over the periods under consideration. Therefore, transaction costs are also not included in the model calculation.

## Example simulation

The following key data were taken into account in the portfolio simulation:

- Start date: 01.01.1963
- Withdrawal rate: 5.0
- No purchases or sales during the year.

The development of the portfolio shown here is illustrated with a starting value of € 100,000 in Table 2.

**Table 2:** Exemplary portfolio development with 50% stocks and 50% bonds

Year	Return FTS03 (%)	Return REXP (%)	Inflation (%)	Withdrawal value at the beginning of the year (€)	Year-End Portfolio-value (€)
1963	14.20	5.37	3.00	5000	104,296
1964	6.86	5.25	2.40	5150	105,149
1965	−12.41	2.90	3.20	5274	95,125
1966	−13.37	1.97	3.30	5442	84,571
1967	49.90	10.31	1.90	5622	102,715
1968	15.42	8.92	1.60	5729	108,789
1969	16.73	0.94	1.80	5820	112,065
1970	−22.54	5.47	3.60	5925	97,081
1971	9.27	8.54	5.20	6139	99,041
1972	16.47	4.07	5.40	6458	102,093
1973	−16.91	3.29	7.10	6806	88,800
1974	2.17	8.23	6.90	7290	85,752
1975	36.28	13.49	6.00	7793	97,362
1976	−3.93	11.15	4.20	8260	92,318
1977	13.34	13.56	3.70	8607	94,969
1978	11.59	3.74	2.70	8926	92,641
1979	−6.21	0.51	4.10	9167	81,095
1980	5.06	3.10	5.40	9542	74,471
1981	4.89	5.07	6.30	10,058	67,621
1982	20.31	18.57	5.20	10,691	67,997
1983	39.83	4.91	3.20	11,247	69,444
1984	12.66	13.19	2.50	11,607	65,314
1985	77.23	10.26	2.00	11,897	76,785
1986	8.89	8.62	−0.10	12,135	70,310
1987	−33.78	6.81	0.20	12,123	50,342
1988	32.61	4.95	1.20	12,147	45,368
1989	38.42	1.61	2.80	12,293	39,695
1990	−14.04	1.41	2.60	12,637	25,349
1991	7.26	11.17	3.70	12,966	13,524
1992	−3.93	13.41	5.00	13,446	82

Source: Own presentation

30 years after the start of the withdrawal phase, assets of around € 82 remain in this scenario. Since it was possible to withdraw the full inflation-adjusted installment in the 30th year after the start of the pension, the portfolio can be described as successful. Accordingly, a portfolio is considered unsuccessful in this analysis if the full withdrawal of the inflation-adjusted rate is not possible before or in the last year of the respective observation period after the start of the pension. Thus, in the example given, the withdrawal of the inflation-adjusted rate would no longer be possible in the 31st year, so the withdrawal rate of 5% and half weighting each of equities and bonds for 35 years at the start of the pension in 1963 is not successful. This “unsuccessful” value enters the analysis as 1/30 because 30 periods can be examined in the data set, covering 35 years.

The underlying dataset on historical returns and inflation rates allows for study periods between 1955 and 2018. The number of records on withdrawal periods is thus as follows:

- 15 years 50 data sets, starting 1955–1969, ending 2004–2018
- 20 years 45 records, starting 1955–1974, ending 1999–2018
- 25 years 40 records, beginning 1955–1979, ending 1994–2018
- 30 years 35 records beginning 1955–1984, ending 1989–2018
- 35 years 30 records, starting 1955–1989, ending 1984–2018

Using this listed scheme, we now simulate portfolio performance for withdrawal periods from 15 to 35 years with seven withdrawal rates ranging from 3 to 9% and five portfolio compositions with an alternating weighting of stocks and bonds in 25% increments.

## Empirical finding

Table 3 shows the results of the simulation.

**Table 3:** Inflation-adjusted Portfolio success rates 1955–2020

	Withdrawal rate as a percentage of the initial portfolio value						
	3%	4%	5%	6%	7%	8%	9%
*100% Stocks*							
15 Years	100	100	100	92	83	69	56
20 Years	100	100	91	77	62	53	47
25 Years	100	95	83	74	60	48	31
30 Years	100	95	76	59	49	41	24
35 Years	100	91	72	56	47	34	25
*75% Stocks/25% Bonds*							
15 Years	100	100	100	100	90	71	60
20 Years	100	100	96	83	66	51	36
25 Years	100	98	90	74	60	45	26
30 Years	100	97	78	65	49	30	19
35 Years	100	94	72	59	47	25	9
*50% Stocks/50% Bonds*							
15 Years	100	100	100	100	92	75	63
20 Years	100	100	100	87	66	51	32
25 Years	100	100	95	74	62	33	12
30 Years	100	100	81	65	43	19	5
35 Years	100	97	72	59	28	16	6
*25% Stocks/75% Bonds*							
15 Years	100	100	100	100	100	77	50
20 Years	100	100	100	91	66	36	11
25 Years	100	100	95	69	40	12	2
30 Years	100	100	78	59	24	3	0
35 Years	100	100	69	41	13	0	0
*100% Bonds*							
15 Years	100	100	100	100	100	71	21
20 Years	100	100	100	98	49	4	0
25 Years	100	100	100	52	5	0	0
30 Years	100	100	73	16	0	0	0
35 Years	100	100	38	3	0	0	0

Source: Own presentation

The results are used to assess the risk of withdrawal rates by providing a link between the recurring, inflation-adjusted withdrawal rate and the historical failure frequency (Cooley et al. 1998, p. 17). The higher the success rate of a withdrawal rate, the lower the number of scenarios that failed in the simulation. “Failed” in this context means that the inflation-adjusted withdrawal was not possible in the last year of the respective period under consideration.

An entitlement to pension payments generally exists at the age of 67. Due to increasing life expectancy, a pension drawdown period of 30 years can be assumed. Consequently, it is primarily the results with a reference period of more than 30 years that are relevant.

Looking at the results, it can be seen immediately that the success rates decrease as the withdrawal rate increases. Thus, the success rate of the 3% withdrawal rate is 100% in each withdrawal period and in each portfolio constellation presented. Consequently, in all historical scenarios, the withdrawal rate of 3% per year, adjusted for inflation, would not once have resulted in the assets being depleted before the end of the withdrawal period. If the withdrawal rate is increased to 4%, this picture changes slightly. For bond shares of 100 to 75%, historically all scenarios are still successful, but the success rate in the withdrawal periods of 25 to 35 years decreases as the equity share becomes larger. For example, at a 4% withdrawal rate over 25 years, only 95% or 38 out of 40 scenarios are successful when the equity ratio is 100%.

It is also not surprising that the success rates decrease the longer the withdrawal continues. Especially at high withdrawal rates such as 7–9%, the reported success rates at withdrawal periods of 25 to 35 years can be classified as difficult to sustain. Success rates below 60% mean that historically at least 2 out of 5 scenarios failed. If the results on high withdrawal rates are translated into recommendations for action, these turn out to be unambiguous for long-term planning horizons. High withdrawal rates are associated with high risk and are therefore not recommendable. A default risk of 40% and more should deter even risk-tolerant individuals.

Looking at the figures, a general question arises which has often been discussed in research: Which success rate can be considered acceptable at all? Here, opinions vary from a relatively aggressive 75% (Cooley et al. 2003, 2011) to a conservative 95% (Terry 2003). If these estimates are constant for acceptable success rates, removal rates between 4 and 5% can be recommended for a 30-year removal period based on the simulation results, depending on risk tolerance. However, the results for a 5% withdrawal rate show that the highest success rate of 80% is achieved with a half distribution of equities and bonds. Consequently, a static withdrawal rate of 5% is only suitable for risk-tolerant investors.

It is striking that the success rates of bond-only portfolios drop very sharply the longer the withdrawal phase lasts. The results thus give the impression that a withdrawal rate historically either produces very good success rates and thus appears to be recommendable or is simply not sustainable due to low success rates.

For investors who are particularly conservative and plan for the long term, the result for the withdrawal rate of 4% with 35 years of withdrawal and 25% shareholding is worth highlighting. The success rate is 100% and is thus superior to the 50/50 weighting. However, in view of current bond yields, the future maintenance of this maximum safe weighting may at least be doubted.

Since the focus of interpretation, in line with the overarching research question, is on withdrawal periods of 30 years, perhaps the most important result of this study should be pointed out here. Withdrawal of inflation-adjusted 4% from a portfolio consisting half of German equities and half of government bonds is successful over each 30-year period studied. This should be highlighted as particularly relevant in the context of international research, as the much respected 4% rule found its origin in precisely this result of the first study of safe withdrawal rates using U.S. capital market data (Bengen 1994).

**Fig. 1:**
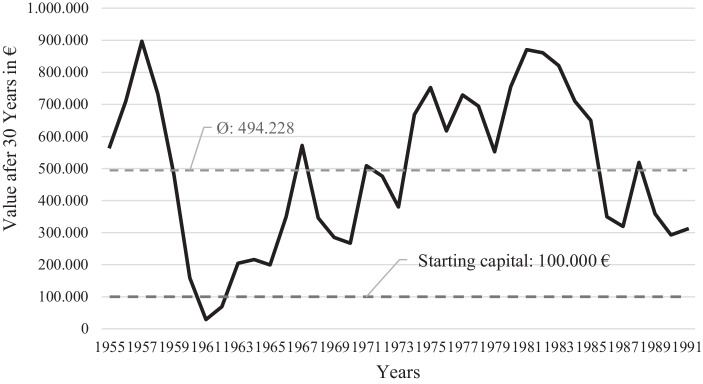
Assets at the end of 30 years of inflation-adjusted withdrawal. (Source: Own presentation based on Kitces 2015)

In summary, the maximum safe withdrawal rate in the existing data set is 4% and a portfolio composition of 50% equities and 50% government bonds is optimal based on an assessment of the average final asset values achieved. Fig. 1 illustrates the resulting final asset values after applying the maximum safe withdrawal rate and optimal portfolio composition. As the figure shows, there are only two data points (1961 & 1962) below the starting capital of € 100,000. If the historical average inflation of 2.6% is taken into account in the valuation, the starting amount of € 100,000 corresponds to a value of around € 100,000 * 1.02630 = € 215,983 after 30 years. Even with this consideration, wealth is accumulated in 30 out of 35 scenarios or about 86% of the observations.

Although the average final asset value can sometimes be described as imposing, there is no question that this unused asset represents an inefficiency of this withdrawal strategy. These assets could have been used for a higher standard of living during the withdrawal period. Reducing this inefficiency is thus a worthwhile task of future research.

## Research limitations

For practical purposes, the 4% rule can be used to derive a reference value from the results. It should be noted that further research questions on the withdrawal strategy must be considered before an ideal withdrawal rate can be derived.

For example, taxes are an important influencing factor, which was not considered in this scientific work. Currently, capital gains are taxed at a flat rate in Germany. Once this changes, further research will be required.

In addition, the average life expectancy in Germany is increasing and thus also the average duration of the withdrawal phase. It is therefore questionable whether a maximum withdrawal period of 35 years is appropriate for all expected lifetimes. An extension of the periods under consideration therefore offers further potential for investigation, also with regard to people who already want to withdraw capital from their portfolio before regular retirement.

The historical novelty of persistently low bond yields deserves increased attention. Modeling and impacting these on future portfolio constellations pose challenges for future retirees. Research in this area can therefore provide answers to the question about asset allocation in times of poor “safe” yields.

The discussion of results also argues for higher equity weightings in terms of higher final asset values. Higher equity weightings inevitably lead to higher volatility. Since the fluctuations of the portfolio constellations are not measured in the study, the conclusion drawn solely on the basis of returns is incomplete and inefficient according to capital market theory. Thus, in order to achieve a more sophisticated evaluation of stock weighting, the measurement of volatility can be an additional decision parameter, which can be elaborated in future research.

## Conclusion and outlook

Research results from an international study with capital market data from 1900 to 2008 show that the maximum safe withdrawal rate for the German capital market in the period under review is 1.14% and that the 4% rule therefore does not apply to Germany (Pfau 2010). In contrast, the data set used for the present study with data from 1955 to 2018 leads to the result that the maximum safe withdrawal rate of 4% is sustainable with a half weighting of German equities and government bonds. This key result can thus be dubbed a significant empirical finding, as the 4% rule is applied in Germany in the empirics. However, it should be emphasized that the result likely originates in the period under consideration.

Political and economic factors, as well as health pandemics such as SARS and viral flu, have led to various stock market crashes since 1955. Despite all crises, the 4%rule holds, so that it can be applied (Boysen-Hogrefe et al. 2020; Popp and Ott 2020). Consequently, the 4% rule can be considered empirically valid due to the long period of analysis considered in the empirical study and the crises considered. It can be assumed that the current COVID-19 pandemic will have no or a small influence on it.

The overall objective of this paper was to determine a safe withdrawal rate taking historical returns of the German capital market into account. In the dataset, the maximum safe withdrawal rate applies to portfolios with 100% bonds, 75% bonds and 50% bonds. The remaining portfolio structure is composed of the respective percentage weighting of equities. At a withdrawal rate of 5%, one in five portfolios already fails.

Determining the optimal weighting of equities and bonds was the second objective of this study. After evaluating the average final asset values achieved, the optimal portfolio composition of the maximum safe withdrawal rate is 50% equities and 50% bonds. For this purpose, first-class government bonds were used as a supposedly safe investment. German stocks were added as the volatile and risky part.

The second and third research questions are related to the advantages of high equity and bond weightings in a diversified retirement portfolio. The advantage of high equity weightings is based on higher average final asset values, which remain at the end of the withdrawal periods. However, high equity weightings are also associated with higher risk. In contrast, no significant advantage can be observed from bond weightings of more than 50%.

The results can be interpreted as a basis for further research on the German capital market. Static strategies have now been overtaken by dynamic approaches. Static withdrawal strategies do not consider the possibility of dynamic consumption behavior. Thus, it can be considered realistic that consumers adjust their consumption and thus the withdrawal rate, e.g., in years of poor market and thus portfolio performance. Consequently, not considering the aforementioned dynamics can lead to inefficiencies. For example, rising consumer prices can lead to a reduction in the standard of living at retirement age.

Monte Carlo simulations could also be used to achieve a higher validity of the research results. Thus, further research is needed to assess a safe withdrawal rate based on the current state of methodology. Nevertheless, the results of the study ultimately showed that equities are also suitable for capital market-based retirement planning in Germany. Transferring the results into the context of declining pension levels, this paper contributes to current and future pension and education policies in Germany. However, in order to establish capital market-supported retirement planning as a solution to the consequences of demographic change, financial literacy is indispensable according to current research findings and should be promoted in terms of education policy.

Although the average final asset value (Fig. 1) can sometimes be described as imposing, there is no question that this unused asset represents an inefficiency of this withdrawal strategy. These assets could have been used for a higher standard of living during the withdrawal period. Reducing this inefficiency is thus a worthwhile task of future research.

## Supplementary Information

Deutsche Version des Artikels
