# Supplementary material for: Empirical determination of sustainable withdrawal rates considering historical yields and inflation rates in Germany
Source: ZVersWiss. 2021 Sep 24;110(2-3):117–32. doi: 10.1007/s12297-021-00504-1 (PMC8475470; doi:10.1007/s12297-021-00504-1)
Supplement: Supplementary file 1 — Deutsche Version des Artikels [file 12297_2021_504_MOESM1_ESM.pdf]

Empirische Ermittlung einer sicheren Entnahmerate für diversifizierte Rentenportfolios unter Berücksichtigung historischer Renditen und Inflationsraten

Einleitung

Niedrige Geburtenraten und eine steigende Lebenserwartung haben in Deutschland zu einem stattfindenden demografischen Wandel geführt. Folglich ist das Rentenniveau netto vor Steuern von 55,0 % im Jahr 1990 auf 48,1 % im Jahr 2018 gesunken (*Bundesministerium für Arbeit und Soziales*, Rentenversicherungsbericht 2018). Aktuell ist für die Entwicklung des Rentenniveaus ab 2030 keine gesetzliche Untergrenze vorgesehen (*Deutsche Rentenversicherung Bund,* 2019). Auch wenn die Rente per Generationenvertrag gesichert ist, wird diese allein nach aktuellen Prognosen für den Großteil der Bevölkerung zum Erhalt des Lebensstandards im Rentenalter nicht reichen. Die skizzierte Gefahr der Altersarmut verschärft sich durch die finanzpolitische Situation am Geld- und Kapitalmarkt weiter, da aufgrund der lockeren Geldpolitik der Europäischen Zentralbank die aktuellen Renditen von Tagesgeld-, Festgeld- oder Girokonten, als auch von Sparbüchern bei um die 0,0 Prozent liegen. Daher investieren deutsche Privathaushalte vermehrt in den Kapitalmarkt und lösen sich zum Teil von traditionellen Anlageformen wie Immobilien, Lebensversicherungen und Bausparverträgen. Nichtsdestotrotz macht der Aktienanteil der Deutschen bei der Altersvorsorge lediglich einen kleinen Teil aus, obwohl zahlreiche fondgebundene Versicherungen und anderweitige Produkte existieren. So liegt der aktuelle Aktionärsanteil der deutschen Bevölkerung bei niedrigen 16,2 Prozent (*Deutsches Aktieninstitut*, 2019).

Unabhängig von der Höhe des zum Renteneintritt vorhandenen Vermögens und dessen Verteilung stellt sich für jeden Rentner eine grundsätzliche Frage: Welcher Betrag oder vielmehr welcher prozentuale Anteil kann regelmäßig aus dem investierten Vermögen entnommen werden? Eine nachhaltige Entnahmestrategie ist notwendig, um das aufgebaute Vermögen nicht innerhalb einer zu erwartenden Lebensdauer aufzubrauchen.

Ziel des Artikels ist die Ermittlung einer angemessenen Entnahmerate vom angesparten Vermögen im Rentenalter, welches mit 67 Jahren beginnt. Folglich widmet sich die Arbeit der Ermittlung einer historisch sicheren Entnahmerate für Rentenportfolios.

Stand der Forschung

Die Mehrheit der publizierten Studien bezieht sich auf den US-amerikanischen Raum. In den USA hat sich zur Beantwortung der Frage der idealen Entnahmerate die sogenannte 4 %-Regel als Orientierungsgröße etabliert. Diese besagt, dass aus Portfolios mit jeweils hälftiger Gewichtung von Aktien und Anleihen 4,0 Prozent des ursprünglichen Portfoliowerts jährlich inflationsangepasst entnommen werden können, ohne dass das Vermögen innerhalb von 30 Jahren aufgebraucht wird (*Bengen, W. P.,* 1994). Historisch haben alle Portfolios in den USA mit 50/50 Verteilung von Aktien und Anleihen jeden 30-Jahreszeitraum bei einer inflationsadjustierten Entnahmerate von 4,0 Prozent überstanden (*Bengen, W. P.,* 1994).

Da die gesetzliche Rentenversicherung in den USA lediglich einen Pfeiler zur Grundsicherung im Alter darstellt, kommt der Vorsorgeberatung durch Externe ein hoher Stellenwert zuteil. So nimmt die Anzahl von Finanzplanern in den USA, welche unter anderem den Titel „Certified Financial Planner“ (CFP) tragen[1], zu. Auch wegen steuerlichen Anreizen zu privater und betrieblicher Altersvorsorge hat das Thema Ruhestandsplanung in den USA einen höheren Stellenwert als in Deutschland. Dies ist einer der Hauptgründe dafür, dass die Forschung zu Entnahmestrategien maßgeblich durch US-amerikanische Studien geprägt ist.

In der Forschung zu Entnahmestrategien und sicheren Entnahmeraten wurde der deutsche Kapitalmarkt bis dato nur in wenigen Untersuchungen berücksichtigt. Aufgrund sinkender Rentenleistungen sowie einer ansteigenden -bezugsdauer wächst das gesellschaftliche Interesse an der Rentenentwicklung. Aus diesem Grund kann dieser Beitrag neben einem großen akademischen, auch einen praxisrelevanten Mehrwert liefern.

Empirie

In dieser Arbeit werden zwei wesentliche Ziele verfolgt. Das erste Ziel ist zu ermitteln, welche maximale Entnahmerate im Kontext historischer Renditen und Inflationsraten

[1] Zum Jahresende 2018 waren weltweit 181 360 zertifizierte Berater im Einsatz, davon 83 106 in den USA und 1 511 in Deutschland, vgl. *Financial Planning Standards Board*, Growth Infographic, 2019. Das Zertifikat wird von Financial Planning Standards Board Ltd. nach umfassenden Prüfungen vergeben.

in Deutschland sicher gewesen ist bzw. ob die 4 %-Regel auch in Deutschland Anwendung finden kann. Als zweites Ziel soll untersucht werden, welche Gewichtung von Aktien und Anleihen für Rentenportfolios unter Berücksichtigung von Rendite und Risiko optimal ist. Die zentralen, aus den formulierten Zielen aufgestellten Leitfragen lauten wie folgt:

1. Welche inflationsangepasste Rate kann regelmäßig aus einem diversifizierten Rentenportfolio bestehend aus deutschen Aktien und Staatsanleihen entnommen werden, ohne dass das Vermögen innerhalb von 30 Jahren vollständig aufgebraucht wird?
2. Welchen Vorteil bieten hohe Anleihegewichtungen in einem diversifizierten Rentenportfolio?
3. Welchen Vorteil bieten hohe Aktiengewichtungen in einem diversifizierten Rentenportfolio?

Diese Untersuchung basiert auf historischen Renditen des Aktien- und Anleihenmarktes in Deutschland. Um eine sichere Entnahmerate zu ermitteln, wird die Entwicklung von Portfolios mit unterschiedlichen Zusammensetzungen und inflationsangepassten Entnahmeraten über Zeiträume von 15 bis 35 Jahren simuliert. Der risikobehaftete Teil des Portfolios wird durch deutsche Aktien, der risikolose Teil durch deutsche Staatsanleihen dargestellt. Das Konzept der Untersuchung wird weitestgehend von der als „Trinity-Studie“ (*Cooley, P. L. et. al.,* 1998) bekannten Untersuchung übernommen.

Datengrundlage des risikobehafteten Teils der Portfolios - Aktien

Für die Simulation der jährlichen Rendite des deutschen Marktportfolios wurde die Frankfurt Top Segment Serie (FTS-Serie) von Stehle/Hartmond und Stehle/Schmidt (*Stehle, R. & Hartmond, A.,* 1991) verwendet. Sie stellt eine solide, historische Datenbasis des Prime Standard als höchstem Börsensegment der an der Frankfurter Wertpapierbörse notierten Unternehmen dar. Die Serie wurde aus verschiedenen offiziellen Quellen nach aufwändiger Prüfung und Verifizierung für die Jahre 1954 bis 2013 zusammengetragen (*Stehle, R., Schmidt, M. H.*, 2015). Die Entwicklungen des Neuen Markts werden in den Berechnungen dieser Serie nicht berücksichtigt. Um dem Anspruch an die Ermittlung der Gesamtrendite aus Sicht eines deutschen Anlegers gerecht zu werden, berücksichtigt diese Datenreihe in der Renditeberechnung neben normalen und Sonderdividenden, Kapitalerhöhungen, Bezugsrechtserlösen und

Nennwertumstellungen auch Körperschaftssteuergutschriften in den Jahren 1977 bis 2000. Da diese Serie im Jahr 2013 endet, kann sie allerdings nicht vollständig als Datengrundlage dienen. Ab 2014 und ebenfalls im vorherigen Zeitraum wird bei der Datenerhebung zusätzlich der Composite DAX (CDAX) als Performanceindex genutzt, da er als Proxy für das deutsche Marktportfolio bzw. den gesamten deutschen Kapitalmarkt interpretiert werden kann. Mit aktuell 445 Werten ist der CDAX deutlich breiter aufgestellt als der DAX.[2]

Datengrundlage des risikolosen Teils der Portfolios – deutsche Staatsanleihen

Der von der Deutsche Börse AG ermittelte deutsche Rentenindex REX setzt sich aus 30 idealtypischen Bundesanleihen mit jeweiligen Laufzeiten von einem bis zehn Jahren zusammen, welche mit ihrem Gewicht im Index multipliziert werden. Da die Berechnung des REX als Kursindex erfolgt, wird in dieser Untersuchung der Performanceindex REXP verwendet, um unter Berücksichtigung von Preisänderungen und Erlösen aus Zinsen die Gesamtrendite des risikolosen Portfolioanteils zu simulieren. Für den REXP sind historische Daten der Bundesbank ab 1967 verfügbar (*Deutsche Bundesbank*, 2019). Für den Zeitraum 1955 – 1966 werden die Umlaufrenditen von Staatsanleihen angesetzt (*Kielkopf, K.*, 1995).**Datengrundlage der Inflationswerte**

Um den Kaufkraftverlust darzustellen, werden vom Statistischen Bundesamt veröffentlichte Inflationsraten in Deutschland verwendet (*Statistisches Bundesamt*, Verbraucherpreisindizes für Deutschland, 2019).

Methodik und Modellannahmen

Im Fokus dieser Arbeit stehen die Ergebnisse zu unterschiedlichen Entnahmeraten, welche anhand historischer Renditen simuliert werden. Die hierzu angewandte Methodik orientiert sich an zwei bestehenden Studien, welche die Forschungsarbeit zu Entnahmestrategien maßgeblich geprägt haben (*Bengen, W. P., 1994 & Cooley, P. L. et al.*, 1998). Seit Veröffentlichung wurde die Methodik etwa durch Monte-Carlo-Simulationen ergänzt, jedoch soll in dieser Arbeit zur besseren Vergleichbarkeit mit Ergebnissen der Trinity-Studie (*Cooley, P. L. et al.*, 1998) die ursprüngliche Methodik rollierender Zeiträume angewandt werden.

Die Zusammensetzung der Portfolios wird mit variablen Risikoprofilen simuliert. Die Staffelung der Portfoliozusammensetzung erfolgt in 25 %-Schritten, startend mit

[2] Stand vom 07.04.2019.

100 % Anleihen und 0 % Aktien bis bin zu 0 % Anleihen und 100 % Aktien. Als Entnahmeraten werden Werte zwischen 3 % und 9 % getestet. Steuerliche Effekte werden in dieser Modellrechnung nicht betrachtet, da dies personenbezogene und individuelle Parameter sind. Der naheliegende Ansatz, die TER eines aktuellen, marktbreiten ETF auf den CDAX als Kostenfaktor für Depotgebühren anzusetzen muss verworfen werden, da im Jahr 1955 keine ETFs existierten und zudem die Verwaltungsgebühren vor der Zeit des Internets höchstwahrscheinlich stark von heutigen Kostenstrukturen abwichen. Auch Transaktionskosten haben sich im Zeitablauf kontinuierlich geändert und sind historisch schwer zu ermitteln. Die Berücksichtigung eines pauschalen Transaktionskostensatzes für die Portfolioumschichtungen stellt sich somit als schwierig dar, auch weil Umschichtungen aufgrund variabler Gewichtungen und Renditen in den Betrachtungszeiträumen schwanken. Aufgrund dessen werden auch Transaktionskosten in der Modellrechnung nicht berücksichtigt.

Beispielsimulation

Bei der Portfoliosimulation wurden folgenden Eckdaten berücksichtigt:

Startzeitpunkt: 01.01.1963

Entnahmerate: 5,0 %

Keine unterjährigen Käufe oder Verkäufe.

Die Entwicklung des hier aufgeführten Portfolios wird mit einem Startwert von 100.000 € in der folgenden *Abbildung* verdeutlicht.

Abbildung 1: Beispielhafte Portfolioentwicklung

Jahr	Rendite FTS03	Rendite REXP	Inflation	Entnahmewert Jahresanfang (€)	Portfoliowert Jahresende (€)
1963	14,20 %	5,37 %	3,00 %	5 000	104 296
1964	6,86 %	5,25 %	2,40 %	5 150	105 149
1965	-12,41 %	2,90 %	3,20 %	5 274	95 125
1966	-13,37 %	1,97 %	3,30 %	5 442	84 571
1967	49,90 %	10,31 %	1,90 %	5 622	102 715
1968	15,42 %	8,92 %	1,60 %	5 729	108 789
1969	16,73 %	0,94 %	1,80 %	5 820	112 065
1970	-22,54 %	5,47 %	3,60 %	5 925	97 081
1971	9,27 %	8,54 %	5,20 %	6 139	99 041
1972	16,47 %	4,07 %	5,40 %	6 458	102 093
1973	-16,91 %	3,29 %	7,10 %	6 806	88 800
1974	2,17 %	8,23 %	6,90 %	7 290	85 752
1975	36,28 %	13,49 %	6,00 %	7 793	97 362
1976	-3,93 %	11,15 %	4,20 %	8 260	92 318
1977	13,34 %	13,56 %	3,70 %	8 607	94 969
1978	11,59 %	3,74 %	2,70 %	8 926	92 641
1979	-6,21 %	0,51 %	4,10 %	9 167	81 095
1980	5,06 %	3,10 %	5,40 %	9 542	74 471
1981	4,89 %	5,07 %	6,30 %	10 058	67 621
1982	20,31 %	18,57 %	5,20 %	10 691	67 997
1983	39,83 %	4,91 %	3,20 %	11 247	69 444
1984	12,66 %	13,19 %	2,50 %	11 607	65 314
1985	77,23 %	10,26 %	2,00 %	11 897	76 785
1986	8,89 %	8,62 %	-0,10 %	12 135	70 310
1987	-33,78 %	6,81 %	0,20 %	12 123	50 342
1988	32,61 %	4,95 %	1,20 %	12 147	45 368
1989	38,42 %	1,61 %	2,80 %	12 293	39 695
1990	-14,04 %	1,41 %	2,60 %	12 637	25 349
1991	7,26 %	11,17 %	3,70 %	12 966	13 524
1992	-3,93 %	13,41 %	5,00 %	13 446	82

Quelle: Eigene Darstellung.

Die nachfolgende *Abbildung* stellt die wesentlichen Ergebnisse der Simulation dar.

Abbildung 2: Inflationsadjustierte Portfolioerfolgsquoten

	Entnahmerate als Prozentsatz des anfänglichen Portfoliowertes						
	**3 %**	**4 %**	**5 %**	**6 %**	**7 %**	**8 %**	**9 %**
**100 % Aktien**							
15 Jahre	100	100	100	92	82	68	56
20 Jahre	100	100	93	80	64	56	49
25 Jahre	100	95	83	73	58	45	30
30 Jahre	100	94	74	57	51	43	26
35 Jahre	100	90	70	57	47	33	23
**75 % Aktien/25 % Anleihen**							
15 Jahre	100	100	100	100	90	70	58
20 Jahre	100	100	96	84	69	53	38
25 Jahre	100	98	90	73	58	43	28
30 Jahre	100	97	77	63	51	31	20
35 Jahre	100	93	70	57	47	23	10
**50 % Aktien/50 % Anleihen**							
15 Jahre	100	100	100	100	92	74	64
20 Jahre	100	100	100	87	69	53	33
25 Jahre	100	100	95	73	60	30	13
30 Jahre	100	100	80	63	46	20	6
35 Jahre	100	97	70	57	27	13	3
**25 % Aktien/75 % Anleihen**							
15 Jahre	100	100	100	100	100	76	52
20 Jahre	100	100	100	91	69	38	11
25 Jahre	100	100	95	68	38	13	3
30 Jahre	100	100	77	57	17	3	0
35 Jahre	100	100	67	40	13	0	0
**100 % Anleihen**							
15 Jahre	100	100	100	100	100	74	22
20 Jahre	100	100	100	98	51	4	0
25 Jahre	100	100	100	50	5	0	0
30 Jahre	100	100	71	14	0	0	0
35 Jahre	100	100	33	3	0	0	0

Quelle: Eigene Berechnung. Darstellung in Anlehnung an *Cooley, P. et al.*, 1998.

Die Ergebnisse dienen der Bewertung des Risikos von Entnahmeraten, da sie eine Verbindung zwischen der wiederkehrenden, inflationsadjustierten Entnahmehöhe und der historischen Ausfallhäufigkeit herstellen. Je höher die Erfolgsquote einer Entnahmerate ausfällt, desto geringer ist die Anzahl an Szenarien, die in der Simulation gescheitert sind. „Gescheitert“ bedeutet in diesem Kontext, dass die inflationsadjustierte Entnahme im letzten Jahr des jeweiligen Betrachtungszeitraums nicht möglich war.

In der Ergebnisinterpretation soll den Ergebnissen zu Entnahmephasen über 30 Jahre gesteigerte Aufmerksamkeit gewidmet werden, da ein Anspruch auf die Regelaltersrente mit Erreichen des 67. Lebensjahres besteht und eine anschließende Rentenbezugszeit von 30 Jahren aufgrund steigender Lebenserwartung zunehmend als realistisch bewertet werden kann.[3]

Bei Betrachtung der Ergebnisse lässt sich unmittelbar feststellen, dass die Erfolgsquoten sinken, je weiter die Entnahmerate steigt. So liegt die Erfolgsquote der Entnahmerate von 3 % in jedem Entnahmezeitraum und bei jeder dargestellten Portfoliokonstellation bei 100 %. Folglich hätte die Entnahme von jährlich inflationsadjustierten 3 % in allen historischen Szenarien nicht ein einziges Mal dazu geführt, dass das Vermögen vor Ablauf der Entnahmephase aufgebraucht wurde. Wird die Entnahmerate auf 4 % gesteigert, ändert sich dieses Bild geringfügig. Bei Anleiheanteilen von 100 % bis 75 % sind historisch noch alle Szenarien erfolgreich, jedoch sinkt die Erfolgsquote in den Entnahmezeiträumen von 25 bis 35 Jahren, je größer der Aktienanteil wird. So sind z. B. bei einer Entnahmerate von 4 % über 25 Jahre nur noch 95 % oder 38 von 40 Szenarien erfolgreich, wenn die Aktienquote bei 100 % liegt.

Es ist zudem wenig verwunderlich, dass die Erfolgsquoten sinken, je länger die Entnahme andauert. Besonders bei hohen Entnahmeraten wie 7 – 9 % sind die ausgewiesenen Erfolgsquoten zu Entnahmezeiträumen von 25 bis 35 Jahren als schwer tragbar zu klassifizieren. Erfolgsquoten unter 60 % bedeuten, dass historisch mindestens 2 von 5 Szenarien gescheitert sind. Überführt man die Ergebnisse zu hohen Entnahmeraten in Handlungsempfehlungen, fallen diese für langfristige Planungshorizonte eindeutig aus. Hohe Entnahmeraten gehen mit einem hohen Risiko einher und sind deshalb nicht zu empfehlen. Ein Ausfallrisiko von 40 % und mehr sollte selbst risikotolerante Personen abschrecken.

Grundsätzlich stellt sich die bereits oft in der Forschung diskutierte Frage, welche Erfolgsquote überhaupt als akzeptabel bezeichnet werden kann. Hierbei weichen die Meinungen von relativ aggressiven 75 % (*Cooley, P. L. et al.* 2003 & 2011) bis hin zu konservativen 95 % (*Terry, R. L.*, 2003) ab. Folgt man diesen Einschätzungen zu akzeptablen Erfolgsquoten, können anhand der Simulationsergebnisse für einen Entnahmezeitraum von 30 Jahren je nach Risikotoleranz Entnahmeraten zwischen 4 % und 5 % empfohlen werden. Die Ergebnisse zu einer 5 % Entnahmerate zeigen allerdings,

[3] Nach aktueller Gesetzgebung steigt das Eintrittsalter fürdie Regelaltersrente zum Jahr 2029 auf 67 Jahre.

dass die höchste Erfolgsquote mit 80 % bei einer hälftigen Verteilung von Aktien und Anleihen liegt. Demzufolge eignet sich eine statische Entnahmerate von 5 % nur für risikotolerante Anleger.

Auffällig ist, dass die Erfolgsquoten von reinen Anleiheportfolios sehr stark abfallen, je länger die Entnahmephase andauert. Die Ergebnisse erwecken somit den Eindruck, dass eine Entnahmerate historisch entweder sehr gute Erfolgsquoten produziert und damit empfehlenswert scheint, oder aber aufgrund niedriger Erfolgsquoten schlicht als nicht nachhaltig zu bewerten ist.

Für besonders konservative und langfristig planende Anleger ist das Ergebnis zur Entnahmerate von 4 % bei 35 Jahren Entnahme und 25 % Aktienanteil hervorzuheben. Die Erfolgsquote liegt bei 100 % und ist damit der 50/50 Gewichtung überlegen. In Anbetracht aktueller Anleiherenditen darf die zukünftige Erhaltung dieser maximal sicheren Gewichtung jedoch zumindest angezweifelt werden.

Da der Interpretationsschwerpunkt gemäß der übergeordneten Forschungsfrage auf Entnahmezeiträumen von 30 Jahren liegt, sei an dieser Stelle auf das vielleicht wichtigste Ergebnis dieser Untersuchung hingewiesen. Die Entnahme von inflationsangepassten 4 % aus einem Portfolio, welches hälftig aus deutschen Aktien und Staatsanleihen besteht, ist über jeden untersuchten Zeitraum von 30 Jahren erfolgreich. Dies ist im Kontext internationaler Forschung als besonders relevant herauszustellen, da die viel beachtete 4 %-Regel ihren Ursprung in genau diesem Ergebnis der ersten Untersuchung zu sicheren Entnahmeraten mit US-amerikanischen Kapitalmarktdaten gefunden hat (*Terry, R. L.*, 2003).

Resümierend ist schließlich festzuhalten, dass die maximal sichere Entnahmerate im vorhandenen Datensatz bei 4 % liegt und eine Portfoliozusammensetzung von 50 % Aktien und 50 % Staatsanleihen nach Bewertung der durchschnittlich erreichten Vermögensendwerte optimal ist. Die folgende *Abbildung* veranschaulicht die entstandenen Vermögensendwerte nach Anwendung der maximal sicheren Entnahmerate und optimaler Portfoliozusammensetzung.

Abbildung 3: Vermögenswerte am Ende von 30 Jahren inflationsadjustierter Entnahme

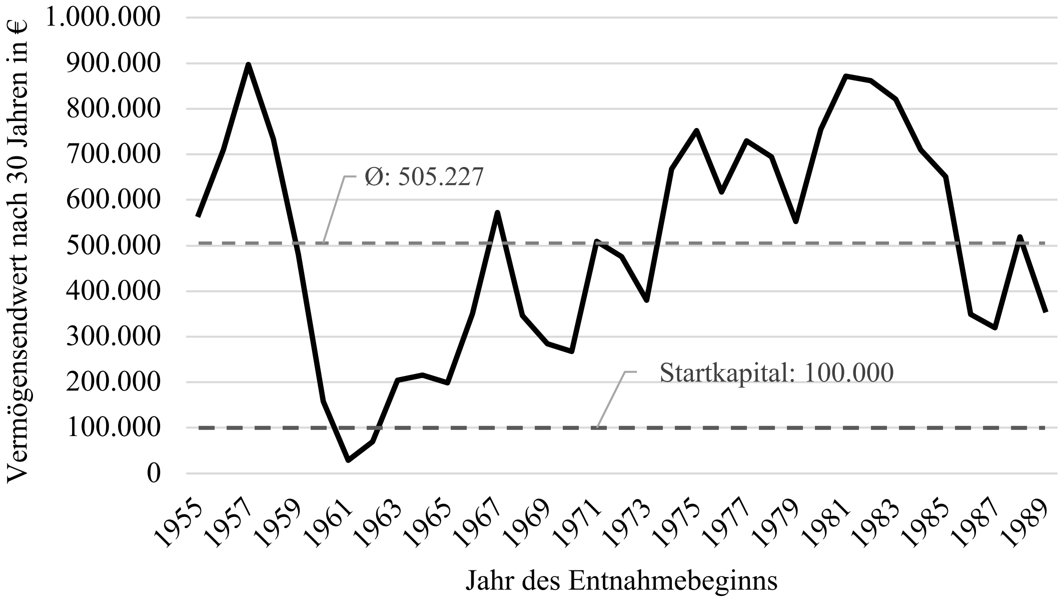

Quelle: Eigene Darstellung in Anlehnung an *Kitces, M. E.*, 2015.

Wie die Abbildung visualisiert, liegen lediglich zwei Datenpunkte (1961 & 1962) unter dem Startkapital von 100.000 €.

Fazit und Ausblick

Forschungsergebnisse einer internationalen Studie mit Kapitalmarktdaten von 1900 bis 2008 zeigen auf, dass die maximale und sichere Entnahmerate für den deutschen Kapitalmarkt im Betrachtungszeitraum bei 1,14 % liegt und die 4 %-Regel für Deutschland somit keine Anwendung findet (*Pfau, W. D.*, 2010). Hingegen führt der für die vorliegende Studie verwendete Datensatz mit Daten von 1955 bis 2018 zum Ergebnis, dass die maximal sichere Entnahmerate von 4 % bei einer hälftigen Gewichtung von deutschen Aktien und Staatsanleihen nachhaltig ist. Dieses zentrale Ergebnis kann somit als wesentlicher empirischer Befund bezeichnet werden, da in der Empirie die 4 %-Regel in Deutschland Anwendung findet. Folglich sollte dieser Befund bei der Umsetzung der privaten Altersvorsorge kritisch berücksichtigt werden. Jedoch ist zu unterstreichen, dass dieses Ergebnis seinen Ursprung aller Wahrscheinlichkeit nach im betrachteten Untersuchungszeitraum hat.

Politische und wirtschaftliche Faktoren, als auch Pandemien wie SARS und die Virusgrippe haben seit 1955 zu diversen Börsencrashs geführt. Da die 4 %-Regel im Datensatz jedoch zum Trotz aller historischen Krisen besteht, kann der empirische Befund aufgrund des langen Betrachtungszeitraums sowohl als valide als auch krisensicher bezeichnet werden. Es ist deshalb davon auszugehen, dass auch der derzeit kursierende Coronavirus keinen Einfluss auf die 4 %-Regel nehmen wird.

Das übergeordnete Ziel dieser Arbeit war die Ermittlung einer sicheren Entnahmerate unter Berücksichtigung historischer Renditen des deutschen Kapitalmarkts. Die maximal sichere Entnahmerate gilt im Datensatz für Portfolios mit 100 % Anleihen, 75 % Anleihen und 50 % Anleihen. Die verbleibende Portfoliostruktur setzt sich aus der jeweiligen prozentualen Aktiengewichtung zusammen. Bei einer Entnahmerate von 5 % scheitert bereits jedes fünfte Portfolio.

Die Ermittlung der optimalen Gewichtung von Aktien und Anleihen war das zweite Ziel dieser Untersuchung. Nach Bewertung der durchschnittlich erreichten Vermögensendwerte liegt die optimale Portfoliozusammensetzung der maximal sicheren Entnahmerate bei 50 % Aktien und 50 % Anleihen. Hierzu wurden erstklassige Staatsanleihen als vermeintlich sicheres Investment herangezogen. Als volatiler und risikobehafteter Teil wurden deutsche Aktien beigemischt.

Die zweite und dritte Forschungsfrage zielen auf die Vorteile hoher Aktien- und Anleihegewichtungen in einem diversifizierten Rentenportfolio ab. Der Vorteil hoher Aktiengewichtungen begründet sich in durchschnittlich höheren Vermögensendwerten, welche am Ende der Entnahmezeiträume übrigbleiben. Allerdings geht mit hohen Aktiengewichtungen auch ein höheres Risiko einher. Ein nennenswerter Vorteil aus Anleihegewichtungen von über 50 % lässt sich hingegen nicht feststellen.

Die Ergebnisse können als Grundlage für weitere Forschung zum deutschen Kapitalmarkt interpretiert werden. Statische Strategien wurden mittlerweile von dynamischen Ansätzen überholt. Statische Entnahmestrategien berücksichtigen nicht die Möglichkeit dynamischen Konsumverhaltens. So ist es als realistisch zu bewerten, dass Verbraucher ihren Konsum bei ansteigenden Konsumentenpreisen anpassen, und die Entnahmerate z. B. in Jahren schlechter Markt- und damit Portfolioentwicklung reduzieren. Folglich kann die Nichtberücksichtigung der angesprochenen Dynamik zu Ineffizienzen führen.

Auch bietet es sich an, mit Monte-Carlo-Simulationen eine höhere Validität der Forschungsergebnisse zu erzielen. Weitere Forschung ist also nötig, um eine sichere Entnahmerate nach dem Stand der Forschung zu beurteilen. Wenngleich der durchschnittliche Vermögensendwert der Abbildung drei mitunter als imposant bezeichnet werden kann, steht außer Frage, dass dieses ungenutzte Vermögen eine Ineffizienz dieser Entnahmestrategie darstellt. Dieses Vermögen hätte während der Entnahmedauer für einen höheren Lebensstandard genutzt werden können. Die Reduzierung dieser Ineffizienz ist somit eine lohnenswerte Aufgabe zukünftiger Forschung.

Nichtsdestotrotz haben die Ergebnisse der Untersuchung letztlich gezeigt, dass sich Aktien auch in Deutschland für die kapitalmarktgestützte Ruhestandsplanung eignen. Überträgt man die Ergebnisse in den Kontext des sinkenden Rentenniveaus, so leistet diese Arbeit einen Beitrag zur aktuellen und künftigen Renten- und Bildungspolitik in Deutschland. Um kapitalmarktgestützte Altersvorsorge jedoch als einen Lösungsansatz für die Folgen des demografischen Wandels zu etablieren, ist Financial Literacy nach aktuellen Forschungserkenntnissen unabdingbar und sollte bildungspolitisch gefördert werden.

Weiterführende Quellen:

***Bengen, William P.** (1994): Determining withdrawal rates using historical data, in: Journal of Financial Planning, 7, Heft 4, S. 171–180*

***Bundesministerium für Arbeit und Soziales** (2019): Stichwort: Rentenniveau, verfügbar unter: https://www.bmas.de/DE/Themen/Rente/Rentenlexikon/Functions/glossar.html?cms_lv2=75306&cms_lv3=86064#glossar86064 (Zugriff: 2019-03-29)*

***Cooley, Philip L, /Hubbard, Carl M., Walz, Daniel T.** (1998): Retirement savings: Choosing a withdrawal rate that is sustainable, in: AAII Journal, 20, Heft 2, S. 16–21.*

— (2003): A comparative analysis of retirement portfolio success rates: Simulation versus overlapping periods, in: Financial Services Review, 12, Heft 2, S. 115–129.

— (2003): Does international diversification increase the sustainable withdrawal rates from retirement portfolios?, in: Journal of Financial Planning, 16, Heft 1, S. 74–81.

— (2011): Portfolio success rates: Where to draw the line, in: Journal of Financial Planning, 24, Heft 4, S. 48–60.

***Deutsche Bundesbank** (o.J.): *Zeitreihe REX Performanceindex, verfügbar unter: https://www.bundesbank.de/dynamic/action/de/statistiken/zeitreihen-datenbanken/zeitreihen-datenbank/723452/723452?tsTab=3&tsId=BBK01.WU046A&listId=www_s140_mb05&id=0 (Zugriff: 2019-04-11).*

***Deutsche Rentenversicherung Bund** (2019): Stichwort: Rentenniveau: Wie weit kann das Rentenniveau bis 2030 sinken?, verfügbar unter: https://www.deutsche-rentenversicherung.de/Allgemein/de/Inhalt/Allgemeines/FAQ/Rente/_%20rentenniveau/rentenniveau.html (Zugriff: 2019-07-14).*

***Deutsches Aktieninstitut** (2019): 50 Jahre Aktien-Renditen: Das DAX-Rendite-Dreieck des Deutschen Aktieninstituts, verfügbar unter: https://www.dai.de/files/dai_usercontent/dokumente/renditedreieck/181231%20DAX-Rendite-Dreieck%2050%20Jahre%20Web.pdf (Zugriff: 2019-05-28).*

— (2019): Mehr Aktionäre in Deutschland: Gleichgültigkeit und Missverständnisse überwinden, Frankfurt am Main: Deutsches Aktieninstitut e.V.

— (2019): Aktionärszahlen des Deutschen Aktieninstituts 2018: Zahl der Aktienbesitzer in Deutschland steigt auf 10,3 Millionen, Frankfurt am Main, 06.03.2019, verfügbar unter: https://www.dai.de/de/das-bieten-wir/studien-und-statistiken/statistiken.html?d=634 (Zugriff: 2019-07-14).

***Kielkopf, Klaus** (1995): Performance von Anleiheportefeuilles: Konzepte, Vergleichsmaßstäbe, Leistung von dt. Rentenfonds., Dissertation, Universität Augsburg, 1995.**Kitces, Michael E.** (2015): The Ratcheting Safe Withdrawal Rate – A More Dominant Version Of The 4% Rule?, verfügbar unter: https://www.kitces.com/blog/the-ratcheting-safe-withdrawal-rate-a-more-dominant-version-of-the-4-rule/ (Zugriff: 27.06.19).*

***Pfau, Wade D.** (2010): An international perspective on safe withdrawal rates from retirement savings: The demise of the 4 percent rule?, in: Journal of Financial Planning, 23, Heft 12, S. 52–61.*

***Statistisches Bundesamt** (2019): Verbraucherpreisindizes für Deutschland: Lange Reihen ab 1948, verfügbar unter: https://www.destatis.de/DE/Themen/Wirtschaft/Preise/Verbraucherpreisindex/Publikationen/_publikationen-verbraucherpreisindex.html?nn=206104 (Zugriff: 2019-04-11).*

***Stehle, Richard, Hartmond, Anette** (1991): Durchschnittsrenditen deutscher Aktien 1954-1988, in: Kredit und Kapital, 24, Heft 3, S. 371–411.*

***Stehle, Richard, Schmidt, Martin H.** (2015): Returns on German Stocks 1954-2013, in: Credit and Capital Markets, 48, Heft 3, S. 427–476.*

***Terry, Rory L.** (2003): The relation between portfolio composition and sustainable withdrawal rates, in: Journal of Financial Planning, 16, Heft 5, S. 64–78.*
